# Genetics and Expression Profile of the Tubulin Gene Superfamily in Breast Cancer Subtypes and Its Relation to Taxane Resistance

**DOI:** 10.3390/cancers10080274

**Published:** 2018-08-18

**Authors:** Babak Nami, Zhixiang Wang

**Affiliations:** Department of Medical Genetics, and Signal Transduction Research Group, Faculty of Medicine and Dentistry, University of Alberta, Edmonton, AB T6G 2H7, Canada; namimoll@ualberta.ca

**Keywords:** breast cancer, tubulin, tubulin βIII (TUBB3), taxane resistance, mRNA expression, gene alteration, mutation, H3K4me3

## Abstract

Taxanes are a class of chemotherapeutic agents that inhibit cell division by disrupting the mitotic spindle through the stabilization of microtubules. Most breast cancer (BC) tumors show resistance against taxanes partially due to alterations in tubulin genes. In this project we investigated tubulin isoforms in BC to explore any correlation between tubulin alterations and taxane resistance. Genetic alteration and expression profiling of 28 tubulin isoforms in 6714 BC tumor samples from 4205 BC cases were analyzed. Protein-protein, drug-protein and alterations neighbor genes in tubulin pathways were examined in the tumor samples. To study correlation between promoter activity and expression of the tubulin isoforms in BC, we analyzed the ChIP-seq enrichment of active promoter histone mark H3K4me3 and mRNA expression profile of MCF-7, ZR-75-30, SKBR-3 and MDA-MB-231 cell lines. Potential correlation between tubulin alterations and taxane resistance, were investigated by studying the expression profile of taxane-sensitive and resistant BC tumors also the MDA-MB-231 cells acquired resistance to paclitaxel. All genomic data were obtained from public databases. Results showed that *TUBD1* and *TUBB3* were the most frequently amplified and deleted tubulin genes in the BC tumors respectively. The interaction analysis showed physical interactions of α-, β- and γ-tubulin isoforms with each other. The most of FDA-approved tubulin inhibitor drugs including taxanes target only β-tubulins. The analysis also revealed sex tubulin-interacting neighbor proteins including ENCCT3, NEK2, PFDN2, PTP4A3, SDCCAG8 and TBCE which were altered in at least 20% of the tumors. Three of them are tubulin-specific chaperons responsible for tubulin protein folding. Expression of tubulin genes in BC cell lines were correlated with H3K4me3 enrichment on their promoter chromatin. Analyzing expression profile of BC tumors and tumor-adjacent normal breast tissues showed upregulation of *TUBA1A*, *TUBA1C*, *TUBB* and *TUBB3* and downregulation of *TUBB2A*, *TUBB2B*, *TUBB6*, *TUBB7P* pseudogene, and *TUBGCP2* in the tumor tissues compared to the normal breast tissues. Analyzing taxane-sensitive versus taxane-resistant tumors revealed that expression of *TUBB3* and *TUBB6* was significantly downregulated in the taxane-resistant tumors. Our results suggest that downregulation of tumor βIII- and βV-tubulins is correlated with taxane resistance in BC. Based on our results, we conclude that aberrant protein folding of tubulins due to mutation and/or dysfunction of tubulin-specific chaperons may be potential mechanisms of taxane resistance. Thus, we propose studying the molecular pathology of tubulin mutations and folding in BC and their impacts on taxane resistance.

## 1. Introduction

Breast cancer (BC) is the most common cancer type in women [1]. Among all BC cases, 20–30% present with metastatic or locally advanced disease, and an additional 30% develop recurrence or metastasis [2]. BC tumors are classified into four subtypes known as luminal A, luminal B, HER2-enriched and basal-like based on the expression of 50 marker genes called the PAM50 panel [2,3].

Metastatic breast cancer (MBC) is among the main causes of cancer mortality in Canada [4,5]. Despite advances in treatment in all settings, disease recurrence and progression remain a major obstacle to therapy [6]. Palliative chemotherapy is often used in metastatic BC at some point in the patient’s therapeutic journey. Taxanes (microtubule-stabilizing agents) are frontline chemotherapy for MBC patients that are used typically alone or in combination with other chemotherapy [5,7,8,9,10]. Despite an initial positive response, most metastatic disease cases eventually progress due to the development of drug resistance [11]. Subsequent chemotherapeutic agents such as anthracyclines, antimetabolites (e.g., capecitabine, gemcitabine), and *vinca* alkaloids (e.g., vinorelbine) are clinically used for taxane-resistant MBC, however, the progressive success is still limited.

Microtubules are formed through the polymerization of the heterodimers of α- and β-tubulins [12,13]. The polymerization and depolymerization dynamics of microtubules are of key importance in spindle formation during mitosis [13,14]. Microtubule targeting agents (MTAs) that effectively inhibit tumor cell mitosis still remain amongst the most reliable anti-cancer chemotherapy agents [15]. MTAs disrupt microtubule dynamics, resulting in abnormal mitotic spindles, chromosome misalignment and the perpetual activation of the spindle assembly checkpoint, which eventually leads to cell death by mitotic catastrophe [16,17]. Current MTAs bind to β-tubulins and are divided into two categories: (1) microtubule-stabilizing agents including taxanes, and (2) microtubule-destabilizing agents, including *vinca* alkaloids, colchicine-site binding agents, and the recently developed eribulin [15,17,18,19].

Although taxanes are widely used in the treatment of BC, the rate of intrinsic and acquired resistance to taxanes is high and a big challenge in taxane therapy [13,20]. So far, several factors proposed for taxane resistance including mutations in both α- and β-tubulins [21], changes in β-tubulin isoform content of microtubules [22], overexpression of drug efflux proteins such as ABCB1 [9,13,23,24], and alteration of microtubule-associated protein levels [25]. In addition, some factors such as dysregulation of proteins related to spindle checkpoints [13,26,27], the cell cycle [13,28], and apoptosis have also been implicated in clinical taxane resistance [13,29,30].

In addition to α- and β-tubulin family, which constitute the tubulin dimer, the tubulin superfamily includes γ-, δ-, ε-, η- families. γ-tubulins play key roles in the nucleation of microtubule assembly at the centrosome. The roles of the other members of the tubulin families are still to be clarified. There are at least nine isoforms of α-tubulin in humans, including tubulin α1A (TUBA1A), α1B (TUBA1B), α1C (TUBA1C), α3C (TUBA3C), α3D (TUBA3D), α3E (TUBA3E), α4A (TUBA4A), α4B (TUBA4B), and α8 (TUBA8) [31,32,33]. Currently, little is known regarding the specific functions of the α-tubulin isoforms. The sequences and structural differences among α-tubulin isoforms are not very significant. The C-terminal tails are the most variable domains among the tubulin isoforms [32]. There are nine isoforms of β-tubulin in humans including tubulin β class I (TUBB), β1 class VI (TUBB1), β2A class IIA (TUBB2A), β2B class IIB (TUBB2B), β3 class III (TUBB3), β4A class IVa (TUBB4A), β4B (TUBB4B), β6 class V (TUBB6), and β8 class VIII (TUBB8) [31,32,33]. The amino acid sequences of the isoforms are strongly conserved, differing predominantly within the 15–20 C-terminal residues [22]. Class III β-tubulin is the most divergent of the major β-tubulin isoforms. For example, the βI-, βII-, and βIV-tubulins are approximately 98% homologous to each other, while βIII-tubulin is only 92% homologous with the others [34,35]. βIII-tubulin is highly expressed in neural tissue [36], and has important role in regulation of neuronal microtubule functions. However, other β-tubulin isoforms are also found in neural tissue which suggesting that βIII-tubulin exerts its specific function in conjunction with other isoforms [37]. βIV-tubulin is exclusively found in axonemes of cilia and flagella [38], however, its exact function in formation of the axoneme has yet to be determined. βI-tubulin, which is essential for the formation of the marginal band a specific microtubule structure in blood platelets, is the most divergent tubulin isoform in mammals [39,40]. Absence or mutation in *TUBB1* gene leads to failure of functional platelet formation, and consequently to bleeding disorders [41].

Several β-tubulin isoforms including βI-, βII-, βIII-, and βIVB-tubulins are expressed in human BC cells [13,21,42]. The expression levels of βII-, βIII-, and βIVB-tubulins have been demonstrated to be linked to malignancy. Especially, βIII-tubulin is clearly linked to the poor outcome [35]. Moreover, the expression levels of βII-, βIII-, and βIVB-tubulins have been linked to taxane resistance [15,19,43]. Isotopically, pure βII- and βIV-tubulins have been found to require a higher ratio of bound paclitaxel to induce microtubule stability, implying the insensitivity of these two isoforms to paclitaxel [44]. Both βIII- and βIV-tubulins are overexpressed in MCF-7 cell line, which was selected for resistance to paclitaxel under increasing paclitaxel concentrations [45]. An increased level of βII-tubulin has also been associated with taxane resistance [46]. Furthermore, it has been reported that the mRNA levels of βII-, βIII-, and βIVB-tubulins are significantly up-regulated in paclitaxel- and docetaxel-resistant MCF-7 cells [47]. Among these β-tubulin isoforms, βIII-tubulin is the most intensively studied. However, evidences regarding its role in taxane resistance is very controversial. Several early studies of ovarian carcinomas demonstrated that βIII-tubulin was shown to enhance the rate of tubulin depolymerization, a feature that could account for resistance to taxanes [48,49,50]. Moreover, it has been argued that the overexpression of βIII-tubulin induces paclitaxel resistance by reducing the ability of paclitaxel to suppress microtubule dynamics [51,52]. However, this notion was challenged by subsequent findings. For example, Hari et al. [53] showed that moderate expression of βIII-tubulin under a conditional promoter induced only a weak (less than two-fold) paclitaxel resistance. Furthermore, increased expression of βIII-tubulin was documented in patients treated with drugs acting as direct inhibitors of tubulin polymerization such as *vinca* alkaloids [54]. Even more problematic, high expression of βIII-tubulin was found associated with sensitivity to taxane-based chemotherapy and not resistance in BC [42,55], clear cell ovarian carcinoma [56] and melanoma [57]. A very recent report showed that βIII-tubulin overexpression has a negligible effect on the sensitivity to taxol in cultured cell lines [58]. We have also shown that in MCF-7 cells selected for taxol resistance, the expression level of βIII-tubulin is not elevated but decreased and βII- and βIV-tubulin levels are increased [42]. These evidences suggest that the accumulated data suggest that βIII-tubulin is not a predictive biomarker of taxane resistance, and instead, βIII-tubulin is a pure prognostic biomarker only when its expression is conditioned by a toxic microenvironment [35].

Taken together, the dysregulation of β-tubulin isoforms has been linked to taxane resistance in various cancers, especially in BC. However, the results regarding the role of individual β-tubulin isoforms are very controversial. Most importantly, none of the previous research studied the expression of levels of various β-tubulin isoforms and other tubulin families in the context of various BC subtypes. The aim of our study is to investigate all tubulin isoforms in BC subtypes and taxane resistance. In this study we analyzed genomic data of large BC tumor sample sets and cell lines to determine tubulin-mediated pathway alterations, genetic alteration and mRNA expression of 28 tubulin genes (including 1 pseudogene) encoding α-, β-, γ-, δ- and ε-tubulin isoforms in BC tumor subtypes also in taxane-sensitive and resistant BC tumors and cell line.

## 2. Results

### 2.1. Tubulin-Tubulin Interactions in BC

**Figure 1:**
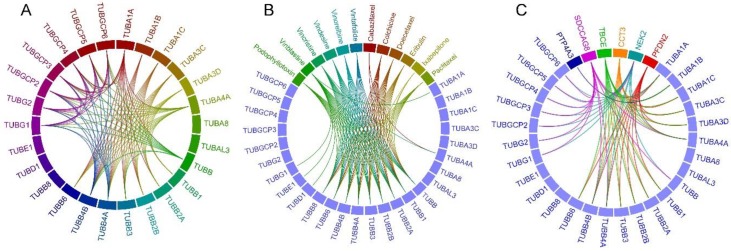
Circle plot of protein-protein and drug-protein interaction in BC tumors. (**A**) Interaction of tubulin isoforms with each other. (**B**) Interaction of FDA-approved drugs with target tubulin isoforms. (**C**) Interaction of frequently altered (minimum of 20% of cases) neighbor genes in tubulin pathways with tubulin isoforms in meta-study BC samples.

To examine the involvement of tubulin families in BC, we first analyzed protein-protein interaction network of 27 tubulin isoforms in BC tumor samples from 4205 meta-study cases. The result showed interaction of TUBA1A and TUBA4A with each other among the α-tubulin isoforms. None of the other α-tubulin isoforms showed interaction with each other. A-tubulin isoforms TUBA1A, TUBA1B, TUBA1C, TUBA3C, TUBA3D, TUBA4A but not TUBA8 and TUBAL3 interacted with the all β-tubulin isoforms TUBB, TUBB1, TUBB2A, TUBB2B, TUBB3, TUBB4A, TUBB4B, TUBB6 except TUBB8 (Figure 1A). TUBA1A and TUBA4A but no other α-tubulins showed interacted with all the γ-tubulin isoforms including TUBG1, TUBG2, TUBGCP2, TUBGCP3, TUBGCP4, TUBGCP5 and TUBGCP6. Among the β-tubulin isoforms, TUBB interacted with TUBB4A and TUBB4B. Also, there was an interaction between TUBB4A and TUBB4B. TUBB, TUBB4A and TUBB4B interact with all the γ-tubulin isoforms. There were interactions between all the γ-tubulins with each other. Interestingly, α-tubulin TUBA8, β-tubulin TUBB8, δ-tubulin TUBD1 and ε-tubulin TUBE1 did not show any interaction with other tubulin isoforms (Figure 1A).

### 2.2. Drug-Tubulin Interactions in BC

To determine tubulin isoforms targeted by anti-cancer chemo-drugs, we performed a drug-target interaction analysis for tubulin isoforms in meta-study samples. Results showed interaction of 12 different FDA-approved drugs with at least one tubulin isoform (Figure 1B). These drugs include cabazitaxel, colchicine, doecetaxel, eribulin, ixabepilone, paclitaxel, podophyllotoxin, vinblastine, vincristine, vindesine, vinorelbine and vintafolide. Cabazitaxel interacts with all the β-tubulins and the α-tubulin isoform TUBA4A. Colchicine, doecetaxel, eribulin, paclitaxel, vindesine, vinorelbine and vintafolide interacted with all the β-tubulin isoforms, but not with other tubulin families. Ixabepilone interacted with the β-tubulin isoforms TUBB, TUBB1 and TUBB4A. Vinblastine interacted with the α-tubulin isoform TUBA1A, all the β-tubulin isoforms, δ-tubulin TUBD1, ε-tubulin TUBE1 and the γ-tubulin isoform TUBAG1, and vincristine interacted with the α-tubulin isoform TUBA4A and all the β-tubulin isoforms (Figure 1B).

### 2.3. Frequently Altered Neighbor Genes

To determine the most frequently altered proteins involved in tubulin pathway in BC, we performed neighbor gene network analysis by filtering the genes with the minimum alteration frequency of 20% in Molecular Taxonomy of Breast Cancer International Consortium (METABRIC) cases. This analysis was to understand which pathway of tubulins is dysregulated in the sample set. Result revealed six highly altered neighbor genes including *CCT3*, *NEK2*, *PFDN2*, *PTP4A3*, *SDCCAG8* and *TBCE* genes. *CCT3* encodes the γ subunit of T-complex protein 1 which is a molecular chaperone and is charged for folding of tubulin [59]. *CCT3* gene was altered in 22% of tumors and was found interacted with all the α- and the β-tubulin isoforms (except TUBB8) (Figure 1C). *NEK2* encodes a centrosomal serine/threonine-protein kinase that regulates centrosome separation, chromatin condensation and spindle formation during mitosis. It is shown linked with breast cancer progression [60,61]. *NEK2* was altered in 22.9% of analyzed tumor samples and found interacted with the α-tubulin isoforms TUBA1A, TUBA4A, the β-tubulin isoforms TUBB, TUBB4A, TUBB4B and all the γ-tubulin isoforms (Figure 1C). *PFDN2* encodes the β subunit of prefoldin, a molecular chaperone complex involving in folding of tubulins [62] and was altered in 21.2% of tumors samples. It was found interacted with the α-tubulin isoforms TUBA1A, TUBA1C, TUBA3C, TUBA3D, TUBA4A and the β-tubulin isoforms TUBB1, TUBB2A, TUBB2B, TUBB3, TUBB4A, TUBB4B and TUBB6 (Figure 1C). *PTP4A3* which was altered in 21.5% of the samples encodes a prenylated protein tyrosine phosphatases and was found interacting with TUBA1B. *SDCCAG8* (serologically defined colon cancer antigen 8) which is localized to the centrioles [63] was altered in 24.5% of cases and found in interaction with the α-tubulins TUBA1A and TUBA4A, the β-tubulins TUBB, TUBB4A and TUBB4B and all the γ-tubulin isoforms. *TBCE* encodes tubulin-specific chaperone E, a molecular chaperon involved in folding of β-tubulins [64]. *TBCE* is altered in 27.8% of the cases and interacts with α-tubulins except TUBA8 and β-tubulins except TUBB8 (Figure 1C).

### 2.4. Tubulin Gene Alterations in BC

**Figure 2:**
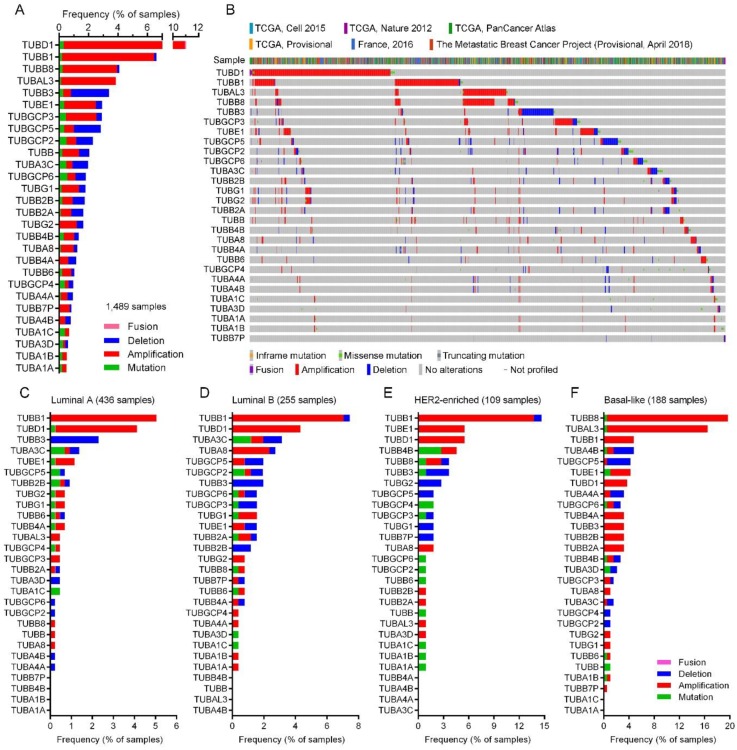
Genetic alteration of tubulin isoforms in meta-study BC tumor samples. (**A**) Frequency of mutation, amplification, deletion and fusion of tubulin isoforms genes. (**B**) Heatmap genetic alteration of tubulin isoforms in each BC tumor samples with at least one alteration. (**C**) Frequency of genetic alteration of tubulin isoforms in the luminal A, (**D**) luminal B, (**E**) HER2-enriched and (**F**) basal-like BC tumors from TCGA, Cell 2015 and TCGA, Nature 2012 studies.

We investigated single nucleotide mutations and copy number alterations (including gene amplification and gene deletion) of 27 tubulin genes and one pseudogene in 6714 BC tumor samples from 4205 meta-study cases. Alterations of tubulin genes in each tumor are shown in Tables S15 and S16. As shown in Figure 2, gene amplification and fusion were the most and the least frequent alteration types respectively, in the tubulin genes. Among the tubulin genes, *TUBD1* (11% of cases) and *TUBB1* (6.6% of cases) were the most frequently altered and amplified, and *TUBA1A* (0.5% of cases) and *TUBA1B* (0.52% of cases) were the least frequently altered tubulin genes. *TUBA3D* was found as the least (0.14% of cases) amplified gene. In the point of view of gene deletion, *TUBB3* was the most (2.57% of cases) deleted gene in the tumors, while *TUBA1A*, *TUBA1B*, *TUBA1C* and *TUBAL3* genes were not deleted in any of the samples (Figure 2).

We also investigated the genetic alterations of tubulin isoforms in PAM50 characterized 436 luminal A, 255 luminal B, 109 HER2-enriched and 188 basal-like subtypes of breast invasive ductal carcinoma tumor samples from The Cancer Genome Atlas (TCGA) studies [65,66]. Heatmap expression plot of PAM50 panel genes for each subtype are shown in Figure S1. Detailed data of gene alterations in each tumor subtype is shown in Tables S17–S24. Results showed that *TUBB1* was the most frequently altered and amplified tubulin gene in the luminal A (5.05% of cases), the luminal B (7.06% of cases) and the HER2-enriched (13.76% of cases) subtypes, while *TUBB8* was the most frequently altered (19.15% of cases) and amplified tubulin gene in the basal-like subtype (Figure 2C–E, Tables S17–S24). Interestingly, *TUBB3* was the most frequently deleted tubulin isoform gene in the luminal A (2.29% of cases), the luminal B (1.96% of cases) and the HER2-enriched (2.75% of cases) subtypes, whereas, the basal-like tumors showed *TUBGCP5* as the most frequent (3.72% of cases) deleted tubulin gene and *TUBB3* was found amplified in 3.19% of the cases (Figure 2C–E, Tables S17–S24).

### 2.5. Tubulin Gene Mutations in BC

**Figure 3:**
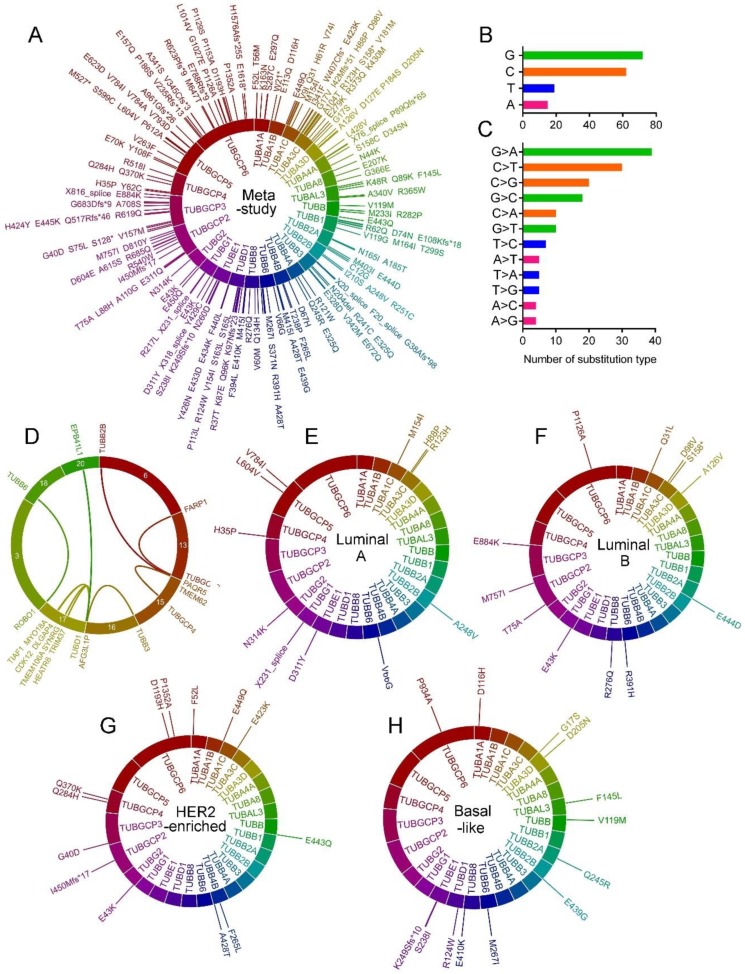
Tubulin gene mutations in BC tumor samples. (**A**) Circle plot of single nucleotide mutations of tubulin isoforms in meta-study BC tumor samples from 4205 BC patients. Layers from inner to outer: gene symbol, scale bars representing protein length, mark bars illustrating the position of mutation, amino acid change mutation identifier. (**B**) The frequency of each mutated nucleotide and (**C**) the type of substitutions. (**D**) Circle plot of tubulin gene fusions. Layers from inner to outer: connection lines illustrating fusions, scale bars representing chromosomes, the fused genes symbols. (**E**) Circle plot of single nucleotide mutations in luminal A, (**F**) luminal B, (**G**) HER2-enriched and (**H**) basal-like BC tumor samples from TCGA, Cell 2015 and TCGA, Nature 2012 studies.

Totally, 336 single nucleotide mutations including missenses, deletions, frame-shifts and splice variants were found in the tubulin genes in meta-study samples. Detail for each mutation is shown in Table S25. *TUBD1* (30 different mutations) and *TUBB4A* (four mutations) was respectively the must and the least frequently mutated tubulin isoforms (Figure 3A). Guanine (G) and cytosine (C) were the most commonly mutated nucleotide type and G > A and C > T were the most frequent substitution type in the all tubulin genes. However, adenine (A) and thymine (T) were rarely mutated (Figure 3B,C). Totally, 14 single case fusions were detected. The most frequently fused tubulin isoform was *TUBD1* (on chromosome 17). *TUBD1* gene was found fused with *MYO18A* (on chromosome 17), *TIAF1* (on chromosome 17), *CDK12* (on chromosome 17), *HEATR6* (on chromosome 17), *DLGAP4* (on chromosome 17), *TRIM37* (on chromosome 17), *TMEM106A* (on chromosome 17), *SYNRG* (on chromosome 17) and *EPB41L1* (on chromosome 20) genes (Figure 3D). Results also showed fusion of *TUBB2B* (on chromosome 6) with *PAQR5* (on chromosome 20), *TUBB3* (on chromosome 16) with *AFG3L1P* (on chromosome 16), *TUBB6* (on chromosome 18) with *ROBO1* (on chromosome 3), *TUBGCP3* (on chromosome 13) with *FARP1* (on chromosome 13) and *TUBGCP4* (on chromosome 15) with *TMEM62* (on chromosome 15) (Figure 3D). Detail for mutations in each BC subtypes is shown in Tables S26–S29. *TUBD1*, *TUBA3C*, *TUBB4B* and *TUBA3D* were the most frequently missense mutated tubulin genes in the luminal A (Figure 3D), the luminal B (Figure 3E), the HER2-enriched (Figure 3F) and the basal-like (Figure 3G) tumors, respectively. No gene fusion was found for tubulin genes in the tumor subtypes.

### 2.6. mRNA Expression of the Tubulin Genes

**Figure 4:**
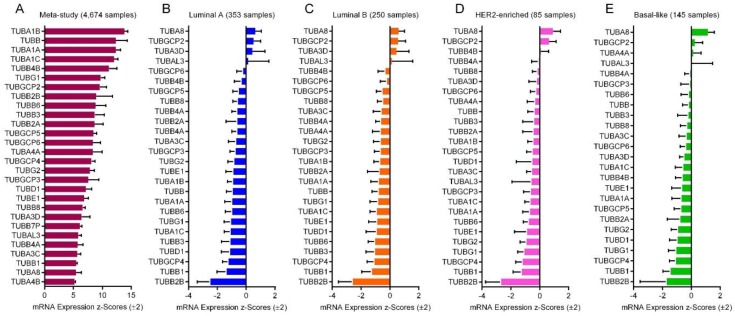
mRNA expression of tubulin isoforms in BC tumors. (**A**) Z-score mRNA expression (RNA-seq v2 RSEM) values of the tubulin isoforms in meta-study BC tumor samples. (**B**) Z-score mRNA expression (microarray) values of the tubulin isoforms in luminal A, (**C**) luminal B, (**D**) HER2-enriched and (**E**) basal-like BC tumor samples from TCGA, Cell 2015 and TCGA, Nature 2012 studies.

We investigated the z-score fold changes RNA-seq expression (v2 RSEM) values for each tubulin gene compared to the expression distribution of each diploid gene in 4674 BC tumor samples from meta-study samples. The expression values for tubulin genes in meta-study samples is shown in Table S30. As result, the α-tubulin isoforms *TUBA1B*, *TUBBA1A* and *TUBA1C* had the highest mRNA expression levels, while *TUBA4B* and *TUBA8* had the lowest expression level among the tubulin isoforms in all the tumor samples (Figure 4A). Among the β-tubulin isoforms, *TUBB* had the highest and *TUBB1* had the lowest mRNA expression levels (Figure 4A).

We also investigated the z-score fold changes microarray RNA expression values of the tubulin genes from 353 luminal A, 250 luminal B, 85 HER2-enriched and 145 basal-like invasive BC tumor samples. The expression values of tubulin genes in each tumor is shown in Tables S31–S34. Result showed significant difference between the BC subtypes on the point of view of mRNA expression levels of all the tubulin genes (ANOVA *P* < 0.001, Figure 4B–E). The α-tubulin isoform *TUBA8* and the β-tubulin isoform *TUBB2B* had respectively the highest and the lowest expression levels in the four tumor subtypes (Figure 4B–E).

### 2.7. Correlation between the Promoter Activity and the Expression of Tubulin Genes

**Figure 5:**
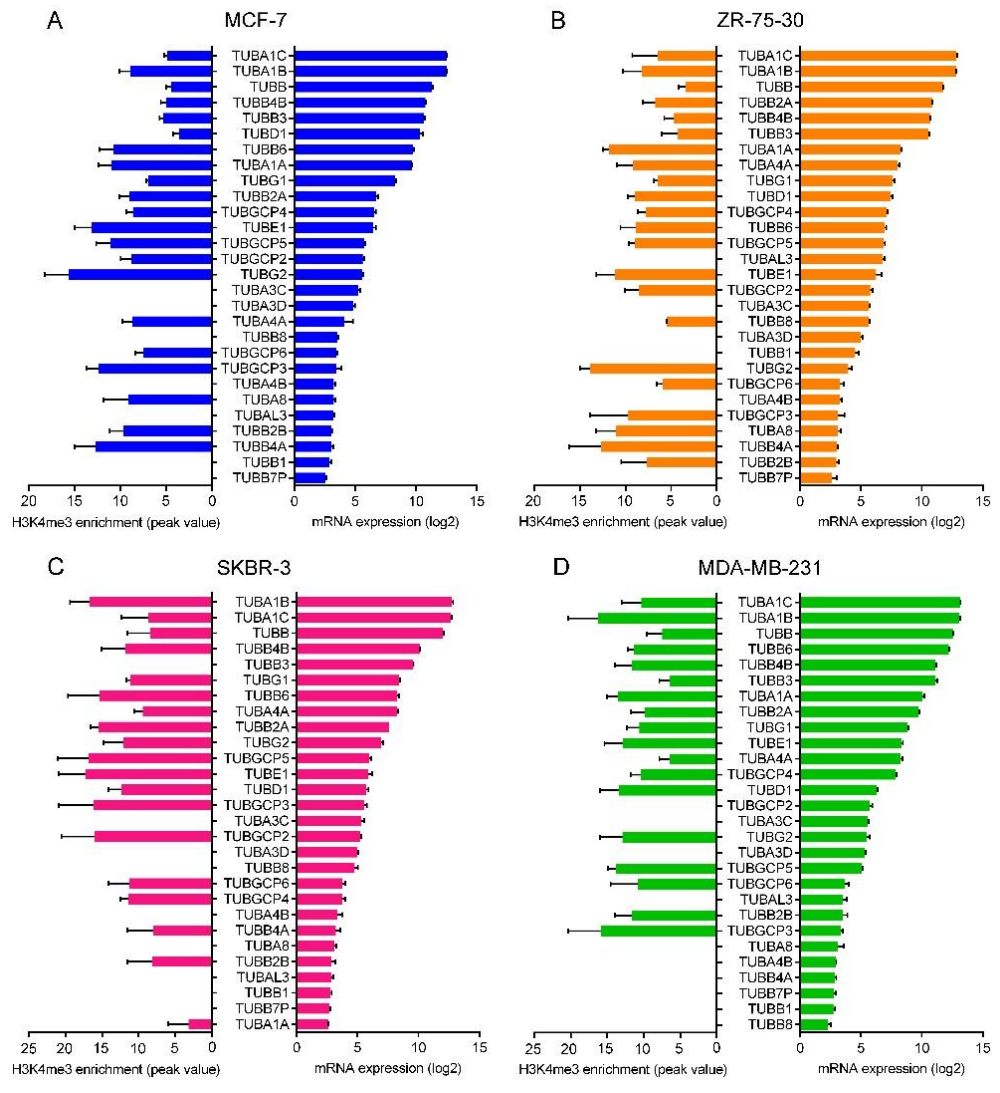
Correlation of promoter activity and expression of the tubulin isoforms. ChIP-seq signal enrichment values of H3K4me3 on the promoter chromatin (left bars) and the mRNA expression (microarray) levels (right bars) of the tubulin genes in (**A**) MCF-7, (**B**) ZR-75-30, (**C**) SKBR-3 and (**D**) MDA-MB-231 cell lines. Data available from GEO datasets with identifiers GSE41313 [67], GSE81714 [72], GSE71327 [73], GSE62966 [74] and GSE49651 [75].

To investigate whether the expression of the tubulin genes is correlated with their promoter activity, we analyzed the enrichment of active promoter mark H3K4me3 on the tubulin promoter and flanking regions in the luminal A BC cell line MCF-7, the luminal B BC cell line ZR-75-30, the HER2-enriched BC cell line SKBR-3, and the basal-like BC cell line MDA-MB-231 cell lines by Cistrome Data Browser. The ChIP-seq signal peaks and values are shown in Figure S2 and Tables S35–S38. We also analyzed the microarray mRNA expression levels of the cell lines obtained from a Gene Expression Omnibus (GEO) dataset. Analyzed ChIP-seq and expression sample series are shown in Table 1. Log2 normalized mRNA expression values are shown in Tables S39–S42. As shown in Figure 5, the α-tubulin isoforms *TUBA1C* and *TUBA1B* and the β-tubulin isoform *TUBB* were found as isoforms with the highest expression levels compared to other isoforms in the four cell lines. *TUBB7P* (pseudogene), *TUBB1* and *TUBA4A* in MCF-7 (Figure 5A), *TUBB7P* (pseudogene), *TUBB2B* and *TUBA4A* in ZR-75-30 (Figure 5B), *TUBA1A*, *TUBB7P* (pseudogene) and *TUBB1* in SKBR-3 (Figure 5C) and *TUBB8*, *TUBB1* and *TUBB7P* (pseudogene) in MDA-MB-231 (Figure 5D) cell lines had the lowest expression levels. H3K4me3 enrichment on the promoter of the tubulin genes in the cell lines revealed that the expressions of tubulin isoforms *TUBA1A*, *TUBA1B*, *TUBA1C*, *TUBA3C*, *TUBA4A*, *TUBA4B*, *TUBA8*, *TUBAL3*, *TUBB*, *TUBB1*, *TUBB2A*, *TUBB3*, *TUBB4B*, *TUBB6*, *TUBB7P*, *TUBB8*, *TUBD1*, *TUBE1*, *TUBG1*, *TUBG2*, *TUBGCP2*, *TUBGCP4* and *TUBGCP5* is correlated with activity of their promoter chromatin. However, there was a dissociation between H3K4me3 enrichment and mRNA expression levels of *TUBA3C*, *TUBA3D*, *TUBB2B*, *TUBB4A* and *TUBGCP3* and *TUBGCP6* genes.

### 2.8. Expression of the Tubulin Genes in Normal Breast and BC Tumors

**Figure 6:**
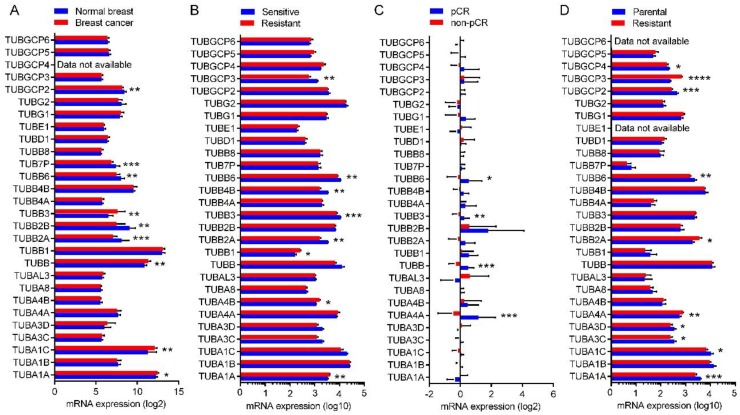
mRNA expression levels of the tubulin genes in normal breast and BC tumor tissues, taxane-sensitive and resistant BC tumor samples. (**A**) Log2 mRNA expression levels of tubulin genes in normal breast biopsies (eight samples) and BC tumors (16 samples) obtained from GEO database ID GSE22796 [68]. (**B**) Log10 mRNA expression levels of the tubulin genes in taxane-sensitive and resistant BC tumors. Data obtained from GEO dataset ID GSE99225. (**C**) Log2 mRNA expression values of the tubulin genes in the tumor biopsies from patients that achieved a pathologic complete response (pCR) (4 samples) and those with residual disease (non-pCR) (13 samples). Data obtained from GEO database ID GSE22513 [69,70]. (**D**) Log10 mRNA expression levels of the tubulin genes in the parental and paclitaxel-resistant MDA-MB-231 cells. Data obtained from GEO database ID GSE12791 [71].

To compare the expression of the tubulin isoforms in normal and tumor tissues of breast, we analyzed microarray expression profile of 16 BC tumors and eight tumor-adjacent normal breast tissues. The data were obtained from GEO dataset GSE22796. Log2 normalized mRNA expression values are shown in Table S43. Results showed the elevated expression of *TUBA1A* (*P* = 0.0201), *TUBA1C* (*P* = 0.0060), *TUBB* (*P* = 0.0020), and *TUBB3* (*P* = 0.0038) and reduced expression of TUBB2A (*P* = 0.0007), *TUBB2B* (*P* = 0.0010), *TUBB6* (*P* = 0.0020), *TUBB7P* (*P* = 0.0001) and *TUBGCP2* (*P* = 0.0020) in BC tumors compared to the normal breast tissues (Figure 6A).

### 2.9. Expression of the Tubulin Genes in Taxane-Sensitive and Resistant BC

To investigate the expression of tubulin genes in taxane-resistant BC tumors we analyzed microarray expression profile of a taxane-sensitive and a resistant tumor tissues without prior exposure to prior treatment. The data obtained from GEO dataset GSE99225 [68]. The sensibility or resistant of the tumor was assessed by quantitative evaluation of tumor viability after *ex vivo* treatment with taxanes using alamar Blue and lactate dehydrogenase release assay. Log10 normalized mRNA expression values are shown in Table S44. As result, the taxane-resistant tumor showed elevated expression levels of *TUBA1A* (*P* = 0.0048), *TUBA4B* (*P* = 0.0335) and *TUBB1* (*P* = 0.0309) and lower expression levels of *TUBB2A* (*P* = 0.0042), *TUBB3* (*P* = 0.0006), *TUBB4B* (*P* = 0.0042), *TUBB6* (*P* = 0.0024) and *TUBCP3* (*P* = 0.0095) in comparison with the taxane-sensitive tumors (Figure 6B).

We also examined the expression array data of tumor biopsies from the patients that achieved a pCR after neoadjuvant taxane-based therapy and those with residual disease (non-pCR). The data obtained from GEO dataset GSE22513 [69]. Log2 normalized mRNA expression values are shown in Table S45. Results showed a downregulated expression of *TUBA4A* (*P* = 0.0008), *TUBB* (*P* = 0.0003), *TUBB3* (*P* = 0.0194) and *TUBB6* (*P* = 0.0290) genes in the tumors from the patients with residual disease (non-pCR) to taxane-based therapy compared to those with pCR (Figure 6C).

For more confirmation, we investigated the expression of the tubulin genes in MDA-MB-231 cells resistant to paclitaxel using data from GEO dataset GSE12791 [71]. Paclitaxel-resistant cells were stablished after treatment with 30 nM paclitaxel for 80 days [71]. Log2 normalized mRNA expression values are shown in Table S46. Results showed decreased expression of *TUBA1A* (*P* = 0.0005), *TUBA1C* (*P* = 0.0243), *TUBA3C* (*P* = 0.0491), *TUBA3D* (*P* = 0.0491), *TUBB6* (*P* = 0.0087), *TUBGCP2* (*P* = 0.0002) and *TUBGCP4* (*P* = 0.0104) and increased expression of *TUBA4A* (*P* = 0.0027), *TUBB2A* (*P* = 0.0158) and *TUBGCP3* (*P* < 0.0001) genes in the paclitaxel-resistant cells compared to the parental cells (Figure 6D).

## 3. Discussion

In this study, by using genomics databases, we analyzed genetic alteration, mRNA expression and the activity of the promoter of 28 tubulin genes (including one pseudogene) in BC subtypes and taxane-resistant BCs. The comparative expression levels of tubulin genes were determined in BC versus normal breast biopsies, luminal A, luminal B, HER2-enriched and basal-like BC tumors and cell lines, taxane-resistant versus taxane-sensitive BC tumors and cell line, as well as the tumors with pCR to taxane treatment compared to non-pCR. Frequency of mutations in each tubulin gene was analyzed in the four BC subtypes. Enrichment of active promoter mark H3K4me3 and mRNA expression were studied for the all tubulin genes in different BC cell lines.

We found that gene amplification was the most frequent alteration in the tubulin genes. Moreover, mRNA downregulation is the most frequent alteration in mRNA transcription levels. In addition, the genetic and expression profile of the tubulin genes were different in the four subtypes of BCs and in the taxane-sensitive and the resistant BC. Protein-protein interaction analysis showed that β-tubulin isoforms interact with α- and γ-tubulin isoforms. This suggest that status of other tubulin isoforms is also important for taxane functions. We found that BC subtypes show different genetic and expression status of tubulin isoforms. We also found that three tubulin-specific chaperons are highly mutated in the BC tumors. Moreover, we observed that expression of tubulin genes is majorly correlated with activity of their promoter chromatin.

One major difference between current research and the most previous research is that we examined the tubulin alterations in various subtypes of BC patients. We found that in most cases, certain alterations only occur in certain subtypes of BC, but not others. Moreover, in some cases, different subtypes of BC showed opposite alterations. For example, while gene amplification were the most frequent alteration types in tubulin genes in all of the BC subtypes, the mostly mutated tubulin isoforms were different among various BC subtypes. *TUBB1* and *TUBD1* were found the most frequent mutated genes in the luminal A, the luminal B and the HER2-enriched subtypes. However, *TUBB8* and *TUBAL3* were the most frequent mutated genes in the basal-like subtype. We also found that the mRNA expression levels of all the other isoforms were significantly different among the BC subtypes.

We also investigated the expression of the tubulin genes in BC cell lines derived from different subtype of tumor cells. These cell lines including luminal A cell line MCF-7, luminal B cell line ZR-75-30, HER2-enriched cell line SKBR-3 and basal-like cell line MDA-MB-231. However, the data are not consistent with the data obtained from patient samples. These inconsistencies suggest that data from just one cell line could not reflect the whole population and thus could not be used as a representative of a specific BC subtype.

Another novel aspect of our research is that in addition to mRNA level we also examined the activity of promoter chromatin of the tubulin genes in BC cell lines. We analyzed ChIP-seq data on the enrichment of active promoter mark H3K4me3 on the promoter and flanking regions of the tubulin genes in the four cell lines. We found that these data are consistent with the data of mRNA level in. There is a positive correlation between H3K4me3 enrichment on the chromatin of the tubulin genes except *TUBA3C*, *TUBA3D*, *TUBB2B*, *TUBB4A* and *TUBGCP3* and *TUBGCP6* genes and their mRNA expression levels in the four BC subtypes. The disassociation of H3K4me3 enrichment and expression levels of the six genes may because of post-transcriptional gene expression regulators that downregulates their transcripts. These data confirm that different mRNA expression levels of the tubulin genes in the four subtypes of BC is due to different chromatin architecture of the BC subtypes. However, some isoforms abrogate this correlation.

Analyzing frequently altered neighbor genes in tubulin pathway showed genetic alteration of six tubulin-interacting proteins in at least 20% of BC tumors. Three of them (*CCT3*, *PFDN2* and *TBCE*) encode chaperone proteins that have key roles in protein folding of tubulins specially α- and β-tubulins. High frequency of alteration of the chaperone genes indicates that misfolding or unfolding of the tubulins may also important roles in BC biology and perhaps in taxane resistance in addition to genetic and expression changes of the tubulins. Tubulin folding play important role in microtubule dynamics [76]. It is possible that misfolded α/β-tubulins could be polymerized and form aberrant microtubules in tumor cells. In these circumstances, taxanes most likely would be unable to interact with target tubulins properly, resulting in taxane resistance. However, there is not yet evidence supporting this notion. It is demonstrated that aberrantly folded tubulin is rapidly degraded by the proteasome leading to tubulin deficiency [77,78]. In addition, accumulation of unfolded/misfolded proteins in the endoplasmic reticulum (ER) can induces ER stress in the tumor cells [79]. It is evidenced that upregulation of survival axis of ER stress signaling can lead taxane resistance [80,81,82]. Taking together, we suggest that improper folding of tubulin proteins is a potential mechanism for taxane resistance in BC.

One major aim of this research was to examine the expression pattern of various β-tubulin isoforms, determine if there is difference between BC and normal cells, and determine if the expression pattern of various β-tubulin isoforms relates to taxane resistance. We investigated the expression of the tubulin genes in 8 normal breast and 16 BC tumors. We found that tumor tissues express significantly higher levels of *TUBB* and *TUBB3* and significantly lower levels of *TUBB2B*, *TUBB6*, and *TUBB7P* pseudogene. 

In terms of taxane resistance, we analyzed expression array data of a taxane-sensitive and a taxane-resistant tumor tissue samples without prior exposure to prior treatment. The sensibility or resistant behavior of the tumor was assessed by quantitative evaluation of tumor viability after *ex vivo* treatment with taxanes using alamar Blue and lactate dehydrogenase release assay. We found that taxane-resistant tumor showed elevated expression levels of *TUBB1* and lower expression levels of *TUBB3*, *TUBB4B*, and *TUBB6* genes when compared with the sensitive tumors. We further examined the expression array data of tumor biopsies from the patients that achieved a pCR after neoadjuvant taxane-based therapy and those with residual disease (non-pCR), we showed a downregulated expression of *TUBB*, *TUBB3* and *TUBB6* genes in the tumors from the patients with residual disease to taxane–based therapy compared to those with pCR. This data is clearly against the dogma that higher βIII-tubulin expression correlates to taxane resistance. Indeed, this dogma has never been supported by solid data [35]. While several early studies of ovarian and lung carcinomas supported this notion [48,49,83], recently more data suggest that higher βIII-tubulin expression does not correlate to taxane resistance. When βIII-tubulin was moderately expressed under a conditional promoter, there was only a weak induction (less than two-fold) of paclitaxel resistance [53]. Even more problematic, in BC [55], clear cell ovarian carcinoma [56] and melanoma [57], studies have reported that high expression of βIII-tubulin was linked to sensitivity to taxane-based chemotherapy and not resistance. A very recent report indicated that βIII-tubulin overexpression has a negligible effect on the sensitivity to taxol in cultured cell lines [58]. We have also shown that in MCF-7 cells selected for taxol resistance, the expression level of βIII-tubulin is not elevated but decreased [42]. Interestingly, in this taxol-resistant cell line, βII- and βIV-tubulin levels are increased [42]. Together, the accumulated data suggest that βIII-tubulin is not a predictive biomarker of taxane resistance. Instead, βIII-tubulin is a pure prognostic biomarker only when its expression is conditioned by a toxic microenvironment [54]. In fact, our data suggest that lower βIII-tubulin expression level actually correlate with taxane resistance, which is consistent with our previous report regarding βIII-tubulin expression in selected docetaxel-resistant MCF-7 cell lines [42].

Breast tumors are highly heterogeneous tissues and contain various types of cells with distinct genetic and epigenetic profile, and response to stressors [84]. Growing evidences demonstrate driving role of cancer stem cells (CSCs) in drug resistance and cancer relapse. CSCs which are also called tumor-initiating cells, are tumor cells with stem cells properties having high plasticity and self-renew capacity [85,86]. CSCs represent only a small fraction of bulk tumor with a subpopulation of 0.1 to 30% of tumor cells depending on the cancer type and the advancement status, however, they have a central role in regulation of tumor niche and overall tumor response to chemotherapy [85,87,88]. In response to changes in tumor microenvironment, CSCs can rapidly switch their biology, differentiate and self-renew in an asymmetric division, survive chemotherapy and initiate new tumors [86,89]. CSCs transition is known as a main cellular level mechanism of drug resistance and cancer relapse. It is demonstrated that high protein expression levels of CSC markers such as ALDH, CD44 and CD133 in tumors is associated with resistance to taxanes in various cancer types [90,91,92]. Indeed, several studies reported permissive outcome of targeting CSCs to overcome taxane resistance (reviewed in [93]). Breast CSCs escape from chemotherapy by different ways, particularly through stemness signaling pathways and altered DNA damage responses [84,94]. Although various molecular mechanisms are suggested for taxane resistance in relation with CSCs, little is known about the mechanism of the association. A recent study showed that CD49f+ CSCs inside triple-negative (ER-/PR-/HER2-) breast cancer tumors are responsible for emergence of taxane resistance [95]. Triple-negative subtype of breast cancer tumors are originated from basal/mesenchymal tissues and are highly enriched for CSCs compared to the other subtypes [88,96,97]. Basal-like breast tumor cells show more stemness properties and plasticity compared to luminal and HER2-enriched cells [84]. Plasticity is an important CSC property that confers tumor the ability to evade from chemotherapy and recurrence. Thus, it is quite expectable that basal-like tumors exhibit CSC features in connection with both stemness and drug resistance abilities. In the current study we found that the genetic profile of tubulin isoforms in basal-like tumors was dramatically different from those in other subtypes. This is a part of genetic hardscape of the tumors and their CSC population. Information about genetic alterations and expression status of tubulin genes in breast CSC is very limited. Taken together, so far evidences suggest breast CSC as a precious cell model for studying the mechanism of taxane resistance. Therefore, investigating tubulin alterations in breast CSC undergoing taxane therapy may be necessary to confirm the roles of tubulin in breast cancer taxane resistance, and to identify evasion cross-ways from chemotherapy.

## 4. Materials and Methods

### 4.1. BC Tumor Genomic Data

Mutation and copy number alteration data from whole exome sequencing, RNA-seq and microarray mRNA expression z-scores of 6714 BC tumor samples from 4205 BC cases (dataset called meta-study) were analyzed. The data were obtained from and The cBioPortal cancer genomics database [98,99] available at http://www.cbioportal.org/index.do. Meta-study dataset includes genomics data from seven independent studies including METABRIC (2509 cases/samples) [100], TCGA, Cell 2015 (818 cases/samples) [65], TCGA, Nature 2012 (825 cases/samples) [66], TCGA, PanCancer Atlas (1084 cases/samples), TCGA, Provisional (1105 cases samples), Mutational profiles of metastatic breast cancer, France, 2016 (216 cases/samples) [101] and The Metastatic Breast Cancer Project, Provisional (157 cases/samples). The pathologic data for each tumor samples and the clinical information of patients from each study are shown in Tables S1–S7 and S8–S14. Microarray or RNA-seq expression profiling data from BC cell lines MCF-7, ZR-75-30, SKBR-3 and MDA-MB-231, BC tumor and adjacent normal breast tissues, taxane-sensitive and resistant BC tissues, BC tumor tissues from patients with pCR and non-pCR, acquired taxane-resistant and parental MDA-MB-231 cell line were obtained from GEO dataset available at https://www.ncbi.nlm.nih.gov/geo. The GEO dataset identifiers are shown in Table 1.

### 4.2. Interaction Network Analysis

Pathway, interaction and drug data were obtained in the context of biological interactions from publicly available pathway and interaction databases Reactome, PANTHER, HPRD, DrugBank, CancerRxGene, KEGG Drugs, pid and Cancer Cell Line Encyclopedia. Interactions and drug data were derived from Pathway Commons and PiHelper web resources respectively. Protein-protein and drug-protein interaction networking was done by Network tool of cBioPortal.

### 4.3. ChIP-Seq Data

ChIP-seq enrichment data of H3K4me3 for MCF-7, ZR-75-30, SKBR-3 and MDA-MD-231 cell lines were obtained from and visualized by Cistrome Data Browser [102] available at http://cistrome.org/db/# using WashU epigenome browser v. 39 available at https://epigenomegateway.wustl.edu/. Raw ChIP-seq data is available from GEO database. The GEO dataset identifiers of ChIP-seq data are shown in Table 1.

### 4.4. Data Analysis and Statistics

GEO microarray expression data was analyzed by Affymetrix Transcriptome Analysis Console (TAC) 3.0 software (Affymetrix Inc., Santa Clara, CA, USA). Heatmaps and circle plots were created by Heatmapper [103] and Circa respectively. Data were statistically analyzed by two-tailed student’s t-test and one-way analysis of variance (ANOVA) using Prism v.6 software (GraphPad Software, La Jolla, CA, USA). Data was presented as mean and SD. *P* < 0.050 was considered as statistically significant.

## 5. Conclusions

Different genetic and expression profile of tubulin genes were found in the four subtypes of BCs and in the taxane-sensitive and the resistant BC. Our results show that tubulin-specific chaperones which are charged for tubulin protein folding are highly mutated in BC. There is a positive correlation between the activities of promoter and enhancers of the tubulin genes and their mRNA expression levels in the four BC subtypes. Our results suggest that downregulation of tumor βIII- and βV-tubulins is correlated with taxane resistance in BC. Thus, higher βIII-tubulin expression level is not correlated with taxane resistance. In fact, our data suggest that lower βIII-tubulin expression levels correlate with taxane resistance. Our results also suggest that mutations and defected tubulin folding pathways may lead to production of aberrant tubulins forming defective microtubules unable to be targeted by taxanes. Thus, investigating the molecular pathology of tubulin gain of function mutations and tubulin folding in BC and their association with taxane resistance is required for future direction.

## Figures and Tables

**Table 1:** GEO dataset and sample identifiers analyzed in this study.

GEO Dataset	GEO Samples	Data Type	Platform	Ref.
GSE41313	GSM1014277, GSM1014278, GSM1014279, GSM1014371, GSM1014372, GSM1014373 GSM1014322, GSM1014323, GSM1014289, GSM1014290, GSM1014291	Log2 RMA signal of array expression profiling for MCF-7, ZR-75-30, SKBR-3 and MD-MB-231 cell lines.	Affymetrix HT HG-U133+ PM Array Plate	[67]
GSE22796	All samples	Log2 quantile normalized signal of beadchip expression profiling for 8 normal breast and 16 BC tumor tissues.	Illumina HumanRef-8 v3.0 expression beadchip	[68]
GSE99225	All samples	Log10 DEVA 1.2 software compute signal intensities of array expression profiling for 2 taxane-sensitive and 2 taxane-resistant BC tumors.	NimbleGen Homo sapiens HG18 expression array	N/A
GSE22513	All samples	Log2 RMA signal of array expression profiling for 4 BC tumors from patients with pathologic complete response (pCR) and 13 BC tumors from patients with non-pCR.	Affymetrix Human Genome U133 Plus 2.0 Array	[69,70]
GSE12791	GSM320837, GSM320838, GSM320841, GSM320842, GSM320845, GSM320846, GSM320849, GSM320850	Log10 MAS 5.0 signal intensity of array expression profiling for paclitaxel-sensitive parental and paclitaxel-resistant MDA-MB-231 cells.	Affymetrix Human Genome U133A Array	[71]
GSE81714	GSM2171845, GSM2171846, GSM2171847, GSM2171848	ChIP-seq signal profiling for H3K4me3 in MCF-7 cell line.	Illumina Genome Analyzer II	[72]
GSE71327	GSM1832644, GSM2029585	ChIP-seq signal profiling for H3K4me3 in ZR-75-30 cell line.	Illumina HiSeq 2500	[73]
GSE62966	GSM1537290, GSM1537285	ChIP-seq signal profiling for H3K4me3 in SKBR-3 cell line.	Illumina HiSeq 2000/2500	[74]
GSE49651	GSM1204472, GSM1204473	ChIP-seq signal profiling for H3K4me3 in MDA-MB-231 cell line.	Illumina HiSeq 1000	[75]

N/A: not available.

## Supplementary Materials

The following are available online at http://www.mdpi.com/2072-6694/10/8/274/s1, Table S1: pathological information of BC tumor samples from METABRIC study, Table S2: pathological information of BC tumor samples from TCGA, Cell 2015 study, Table S3: pathological information of BC tumor samples from TCGA, Nature 2012 study, Table S4: pathological information of BC tumor samples from TCGA, PanCancer Atlas study, Table S5: pathological information of BC tumor samples from TCGA, Provisional study, Table S6: pathological information of BC tumor samples from Mutational profiles of metastatic breast cancer (France, 2016) study, Table S7: pathological information of BC tumor samples from The Metastatic Breast Cancer Project (Provisional, April 2018) study, Table S8: clinical information of BC cases and tumors from METABRIC study, Table S9: clinical information of BC cases and tumors from TCGA, Cell 2015 study, Table S10: clinical information of BC cases and tumors from TCGA, Nature 2012 study, Table S11: clinical information of BC cases and tumors from TCGA, PanCancer Atlas study, Table S12: clinical information of BC cases and tumors from TCGA, Provisional study, Table S13: clinical information of BC cases and tumors from The Mutational profiles of metastatic breast cancer (France, 2016) study, Table S14: clinical information of BC cases and tumors from The Metastatic Breast Cancer Project (Provisional, April 2018) study, Table S15: type of genetic alterations across BC tumor samples from meta-study, Table S16: alteration frequency of tubulin isoform genes (% of samples) in the BC tumor samples from meta-study, Table S17: type of genetic alterations across the luminal A BC tumor samples from TCGA, Cell 2015 and TCGA, Nature 2012 studies, Table S18: alteration frequency of tubulin isoform genes in the luminal A BC tumor samples from TCGA, Cell 2015 and TCGA, Nature 2012 studies, Table S19: type of genetic alterations across luminal B BC tumor samples from TCGA, Cell 2015 and TCGA, Nature 2012 studies, Table S20: alteration frequency of tubulin isoform genes in the luminal B BC tumor samples from TCGA, Cell 2015 and TCGA, Nature 2012 studies, Table S21: type of genetic alterations across HER2-enriched BC tumor samples from TCGA, Cell 2015 and TCGA, Nature 2012 studies, Table S22: alteration frequency of tubulin isoform genes in the HER2-enriched BC tumor samples from TCGA, Cell 2015 and TCGA, Nature 2012 studies, Table S23: type of genetic alterations across basal-like BC tumor samples from TCGA, Cell 2015 and TCGA, Nature 2012 studies, Table S24: alteration frequency of tubulin isoform genes in the basal-like BC tumor samples from TCGA, Cell 2015 and TCGA, Nature 2012 studies, Table S25: detailed tubulin isoform mutations in meta-study cases, Table S26: detailed tubulin isoforms mutations in the luminal A BC tumor samples from TCGA, Cell 2015 and TCGA, Nature 2012 studies, Table S27: detailed tubulin isoforms mutations in the luminal B BC tumor samples from TCGA, Cell 2015 and TCGA, Nature 2012 studies, Table S28: detailed tubulin isoforms mutations in the HER2-enriched BC tumor samples from TCGA, Cell 2015 and TCGA, Nature 2012 studies, Table S29: detailed tubulin isoforms mutations in the basal-like BC tumor samples from TCGA, Cell 2015 and TCGA, Nature 2012 studies, Table S30: mRNA expression values (RNA-seq v2 RSEM) of the tubulin genes in the BC tumors from meta-study samples, Table S31: mRNA expression values (microarray) of the tubulin genes in the luminal A BC tumors from the TCG, Cell 2015 and TCG, Nature 2012 studies, Table S32: mRNA expression values (microarray) of the tubulin genes in the luminal B BC tumors from the TCG, Cell 2015 and TCG, Nature 2012 studies, Table S33: mRNA expression values (microarray) of the tubulin genes in the HER2-enriched BC tumors from the TCG, Cell 2015 and TCG, Nature 2012 studies, Table S34: mRNA expression values (microarray) of the tubulin genes in the basal-like BC tumors from the TCG, Cell 2015 and TCG, Nature 2012 studies, Table S35: normalized H3K4me3 ChIP-seq signal peak values on the promoter regions of the tubulin genes in MCF-7 cell line, Table S36: normalized H3K4me3 ChIP-seq signal peak values on the promoter regions of the tubulin genes in ZR-75-30 cell line, Table S37: normalized H3K4me3 ChIP-seq signal peak values on the promoter regions of the tubulin genes in SKBR-3 cell line, Table S38: normalized H3K4me3 ChIP-seq signal peak values on the promoter regions of the tubulin genes inMDA-MB-231 cell line, Table S39: log2 mRNA expression values (microarray) of the tubulin genes in MCF-7 cell line, Table S40: log2 mRNA expression values (microarray) of the tubulin genes in ZR-75-30 cell line, Table S41: log2 mRNA expression values (microarray) of the tubulin genes in SKBR-3 cell line, Table S42: log2 mRNA expression values (microarray) of the tubulin genes in MDA-MB-231 cell line, Table S43: log2 mRNA expression values of the tubulin genes in 8 normal breast and 16 BC tumors. The data obtained from GEO dataset ID GSE22796, Table S44: Log10 normalized mRNA expression values of the tubulin genes in a taxane-sensitive and a taxan-resistant BC tumor. The data obtained from GEO dataset ID GSE99225, Table S45: log2 normalized mRNA expression values of the tubulin genes in tumor biopsies from the patients that achieved a pathologic complete response (pCR) and those with non-PCR. The data obtained from GEO dataset ID GSE22513, Table S46: Log10 normalized mRNA expression values of the tubulin genes in parental and acquired taxane-resistant MDA-MB-231 cells. The data obtained from GEO dataset ID GSE12791, Figure S1: hieratical heatmap array expression of PAM50 panel genes in (A) luminal A, (B) luminal B, (C) HER2-enriched and (D) basal-like BC tumors. Data obtained from TCGA, Cell 2015 study, Figure S2: ChIP-seq signal peak values of H3K4me3 on the promoter region of the tubulin genes in BC cell lines.
