# Supplementary figures and images for: Genetics and Expression Profile of the Tubulin Gene Superfamily in Breast Cancer Subtypes and Its Relation to Taxane Resistance
Source: Cancers (Basel). 2018 Aug 18;10(8):274. doi: 10.3390/cancers10080274 (PMC6116153; doi:10.3390/cancers10080274)

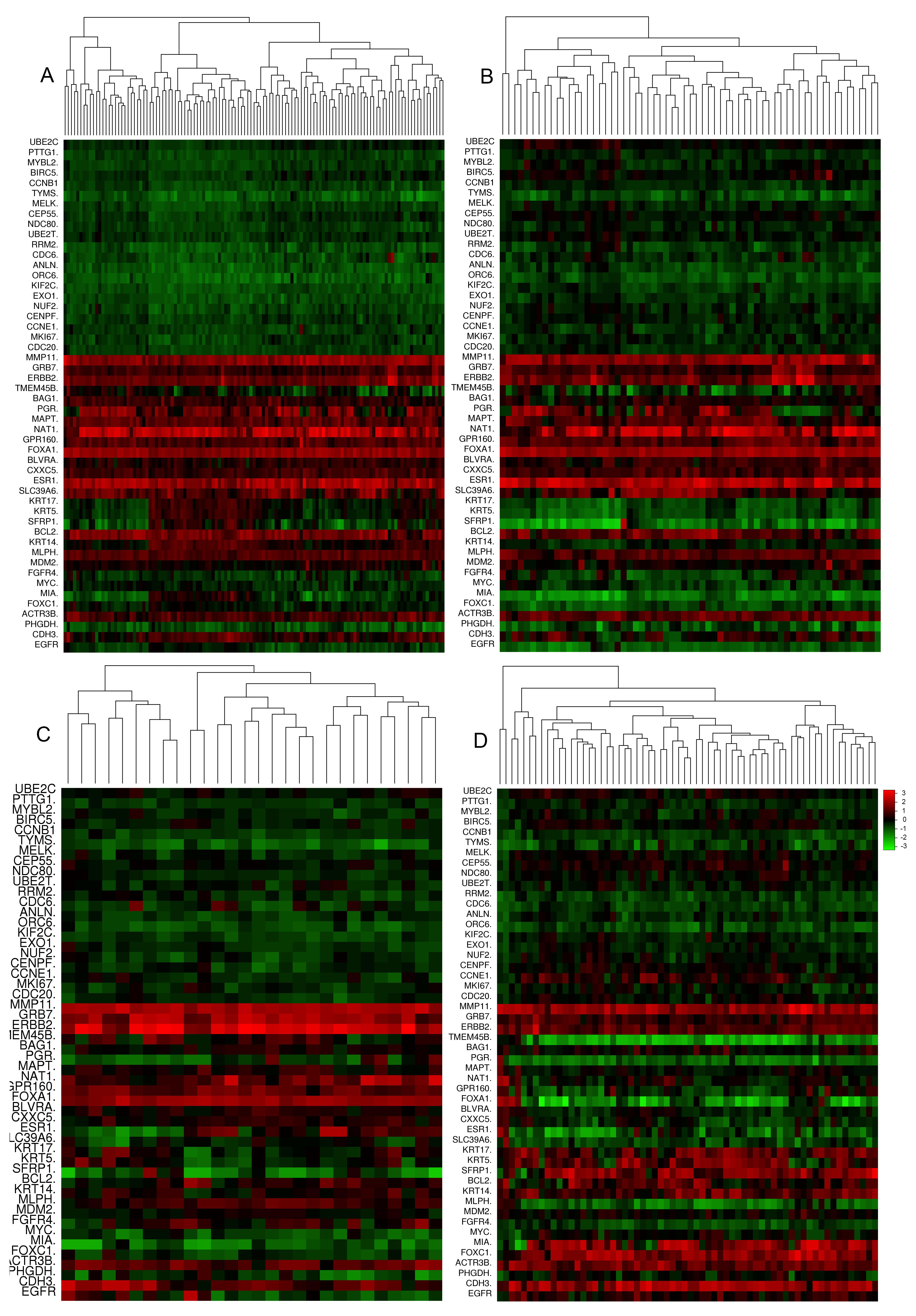

Supplement: Supplementary file 1 [file cancers-10-00274-s001.zip › FigureS1_PAM50.jpg]

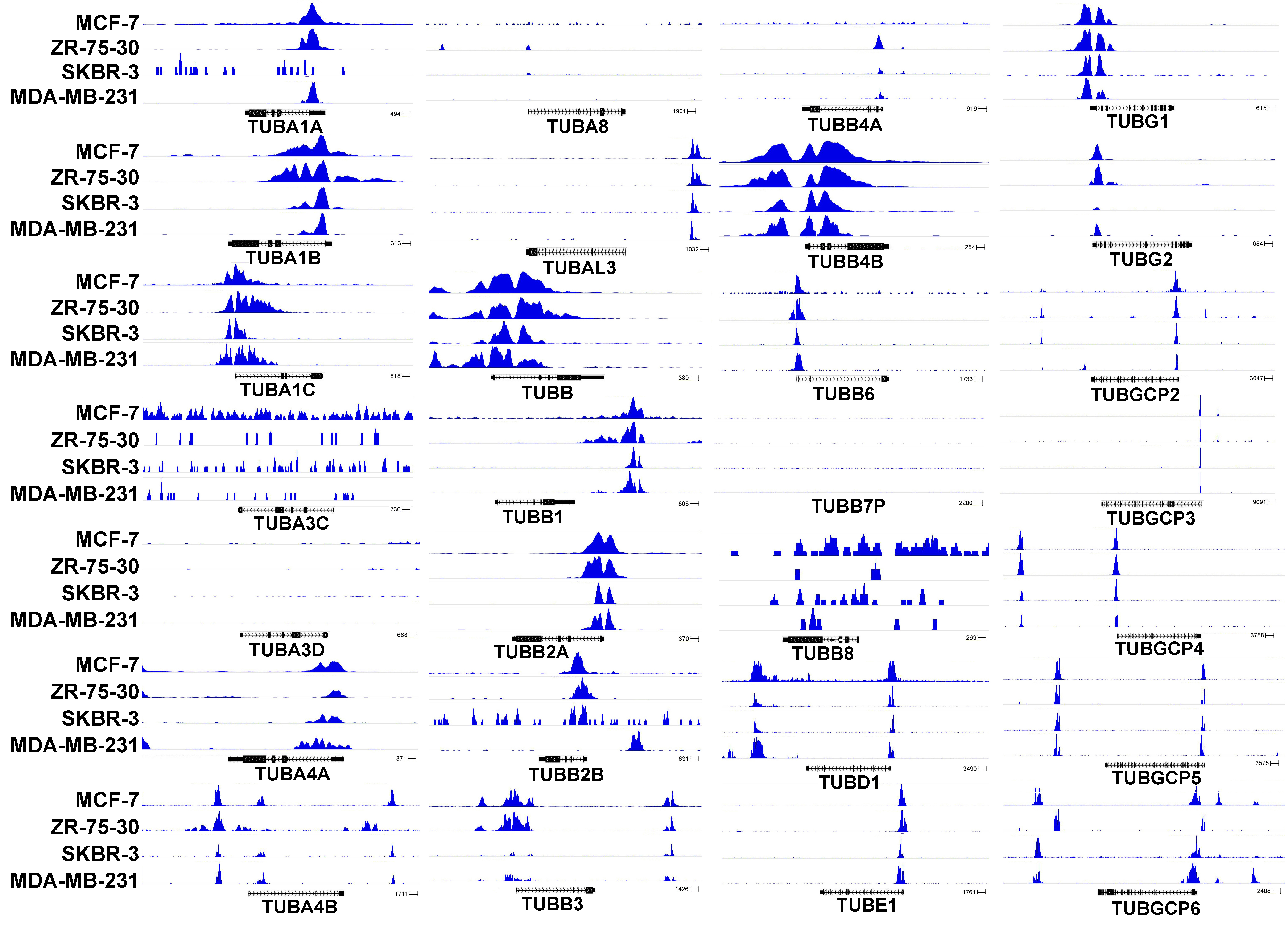

Supplement: Supplementary file 1 [file cancers-10-00274-s001.zip › FigureS2_H3K4me3.jpg]
